# A semi-parametric mixed models for longitudinally measured fasting blood sugar level of adult diabetic patients

**DOI:** 10.1186/s12874-018-0648-x

**Published:** 2019-01-10

**Authors:** Tafere Tilahun Aniley, Legesse Kassa Debusho, Zelalem Mehari Nigusie, Wondwosen Kassahun Yimer, Belay Birlie Yimer

**Affiliations:** 1 Department of Statistics, Jimma University, Jimma, Ethiopia; 2 Department of Statistics, University of South Africa, c/o Christiaan de Wet Road & Pioneer Avenue, Johannesburg, Private Bag X6, Florida 1710 South Africa; 3 Department of Epidemiology and Biostatistics, College of Medicine and Health Sciences, Bahir Dar university, Bahir Dar, Ethiopia; 4 Department of Data science, The university of Mississippi Medical center, 2500 North State, Jackson, 39216 MS USA; 5 Arthritis Research UK Centre for Epidemiology, Division of Musculoskeletal and Dermatological Sciences, The University of Manchester, Manchester, UK

**Keywords:** Diabetes mellitus, Fasting blood sugar, Linear mixed model, Semi-parametric mixed model

## Abstract

**Background:**

At the diabetic clinic of Jimma University Specialized Hospital, health professionals provide regular follow-up to help people with diabetes live long and relatively healthy lives. Based on patient condition, they also provide interventions in the form of counselling to promote a healthy diet and physical activity and prescribing medicines. The main purpose of this study is to estimate the rate of change of fasting blood sugar (FBS) profile experienced by patients over time. The change may help to assess the effectiveness of interventions taken by the clinic to regulate FBS level, where rates of change close to zero over time may indicate the interventions are good regulating the level.

**Methods:**

In the analysis of longitudinal data, the mean profile is often estimated by parametric linear mixed effects model. However, the individual and mean profile plots of FBS level for diabetic patients are nonlinear and imposing parametric models may be too restrictive and yield unsatisfactory results. We propose a semi-parametric mixed model, in particular using spline smoothing to efficiently analyze a longitudinal measured fasting blood sugar level of adult diabetic patients accounting for correlation between observations through random effects.

**Results:**

The semi-parametric mixed models had better fit than the linear mixed models for various variance structures of subject-specific random effects. The study revealed that the rate of change in FBS level in diabetic patients, due to the clinic interventions, does not continue as a steady pace but changes with time and weight of patients.

**Conclusions:**

The proposed method can help a physician in clinical monitoring of diabetic patients and to assess the effect of intervention packages, such as healthy diet, physical activity and prescribed medicines, because individualized curve may be obtained to follow patient-specific FBS level trends.

## Background

Diabetes mellitus is a metabolic disorder of multiple etiology characterized by chronic hyperglycaemia with disturbances of carbohydrate, fat and protein metabolism resulting from defects in insulin secretion, insulin resistance, or both [1]. The long-term effects of untreated diabetes mellitus might results in health complications, such as visual disability and nerve disease [2–5], among others. A person is considered to be diabetic if he or she has fasting blood sugar (FBS) level value of greater than or equal to 7.0 mmol/L (126 mg/dL) or 2-h blood sugar level of greater than or equal to 11.1 mmol/L (200 mg/dL) or glycated hemoglobin (HbA_1_) level of 6.5% or higher [6].

There are three main types of diabetes, namely type 1 diabetes, type 2 diabetes and gestational diabetes. The type 1 diabetes is caused by an auto-immune reaction, in which the patient body defense system attacks the insulin producing beta cells in the pancreas and hence the body can no longer produce the insulin it needs. Whereas in type 2 diabetes, the body is able to produce insulin, however it becomes resistant so that the insulin is ineffective. The type 2 diabetes is characterized by high levels of blood sugar or glucose resulting from defects in insulin production, insulin action, or both. The gestational diabetes is a form of diabetes that appears during pregnancy. It can lead to serious health risks for both the mother and child [7]. The risk factors that are associated with type 1 diabetes include family history of diabetes (diabetes history in one parent or both), infections and other environmental influences such as exposure to a viral illness, the presence of damaging immune system cells, i.e. autoantibodies and dietary factors low vitamin D consumption [8]. Whereas, for type 2 diabetes the risk factors are excess body weight, physical inactivity, poor nutrition, family history of diabetes, past history of gestational diabetes and older age [9]. The risk factors for increase or decrease in fasting blood sugar level of a patient include overweight, family history of diabetes, age, type of diabetes, blood pressure and gender [7]. The focus of this study however is on type 1 and type 2 diabetes.

In year 2015, there were an estimated 415 million adults aged 20–79 years living with diabetes worldwide [10], including 193 million who are undiagnosed. There were approximately 5 million people estimated to have died from diabetes worldwide in the same year, and a majority of these were the result of cardiovascular complications. In Africa Region, the number of adults living with diabetes estimated at 14.2 million whereas in Ethiopia the number is estimated 1 to 10 million in year 2015. The Region has the highest proportion of undiagnosed diabetes, 9.5 million (about 66.7%) of people with diabetes are unaware they have the disease and in Ethiopia there are 500 thousand to 5 million such cases [11, 12].

At the diabetic clinic of Jimma University Specialized Hospital (JUSH), health professionals provide regular follow-up to help people with diabetes live long and relatively healthy lives. Depending on patients conditions, e.g. FBS level, they also provide interventions in the form of counselling to promote a healthy diet and physical activity and prescribing medicines.

The main objective of the current study is to assess the factors that affect the FBS level of adult diabetic patients. In addition to assessing the factors that affect the FBS level over time, we are also interested to estimate the rate of change of FBS profile experienced by patients over time. The change may help to assess the effectiveness of interventions taken by the clinic to regulate FBS level, where rates of change close to zero over time may indicate the interventions are good regulating the level. These changes are determined using first derivatives of penalized regression splines [13, 14].

**Fig. 1:**
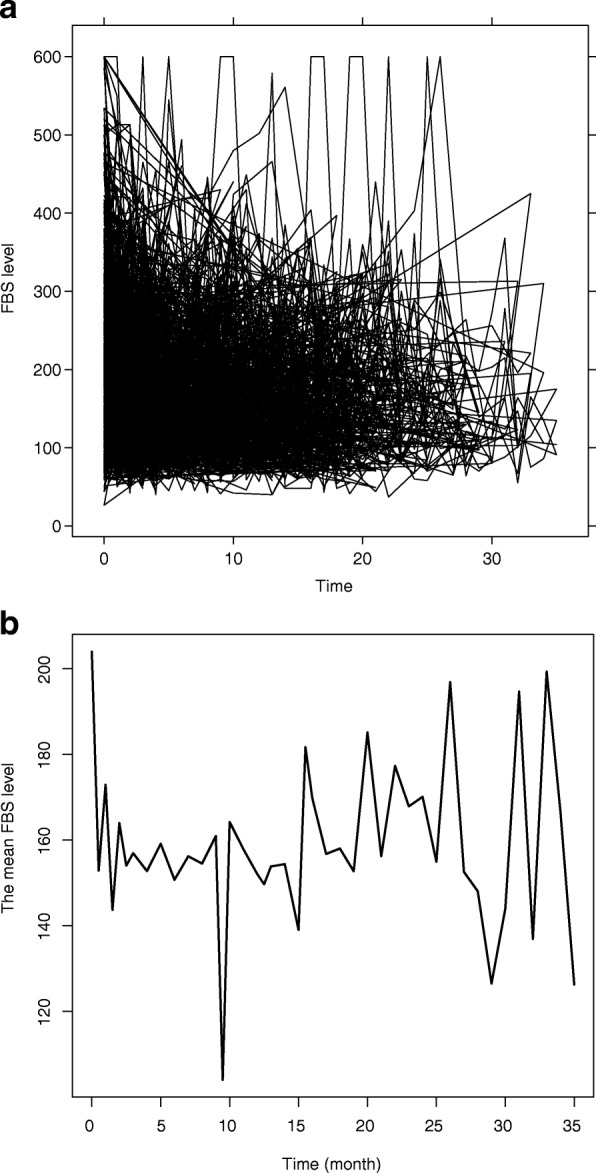
(**a**) individual profile and (**b**) mean profile plots for FBS level of diabetes patients in JUSH, September 2011 - June 2014

The FBS level data of diabetic patients in this study are collected repeatedly over time hence the data have longitudinal time series profiles and the data also have continuous nature. For statistical inferences, therefore, it is necessary to capture properly the form of the evolution of profiles over time. In the analysis of longitudinal data, the mean profile is often estimated by parametric linear mixed effects model, for instance recently Mehari [15] analyzed the FBS level profiles of diabetic patients using parametric linear mixed effects model. However, the individual and mean profile plots of FBS level for diabetic patients (see Fig. 1) are nonlinear and imposing parametric models may be too restrictive and yield unsatisfactory results. In the present paper, we propose a semi-parametric mixed model in particular using spline smoothing [16, 17] to efficiently analyze a longitudinal measured fasting blood sugar level of adult diabetic patients accounting for correlation between observations through random effects. The model assumes that the mean of FBS level is an arbitrary smooth function of time and parametric functions of other covariates. The link between mixed model and smoothing provides a flexible framework for estimating the patient profiles in a data driven way [13].

The rest of the paper is organized as follows. The data, some basic review of variance-covariance structure of the parametric linear mixed model, semi-parametric mixed models and inferences related them are introduced in “Methodology” section. The results from applying these methods on the the study data are discussed in “Results” section. Finally discussion, and conclusions and pointers for future study are given in “Discussion” and “Conclusion” sections respectively.

## Methodology

### Study data

The fasting blood sugar (FBS) level data used in this paper arises from a retrospective study conducted in Jimma University Specialized Hospital (JUSH) diabetic clinic. The hospital is located in Jimma town 352 km to the Southwest of Addis Ababa, the capital of Ethiopia. It is a teaching hospital and gives service to the southwestern part of Oromia region, some part of southern nations and nationalities and Gamella regions of Ethiopia. All diabetic patients aged 18 years or older, who were coming to JUSH diabetic clinic for their regular follow up during periods September 2011 and June 2014 were eligible for this study. During their follow up, patients FBS level along with other characteristics such as weight are measured and recorded in the individual follow up chart. The data in the chart include time (measured in months, where baseline or initial date was given a value 0), patient gender, age, type of diabetes (Type 1 diabetes or Type 2 diabetes) and family diabetes history. The duration between initial and the last recorded visits ranged from one to 36 months. Patients with at least two observations were included in the analyses leading to a total of 534 patients and 4390 observations. Permission of the study was obtained from Postgraduate research office of Jimma University, College of Natural Sciences and JUSH.

### Variance-covariance structures and inference

#### Variance-covariance structures

The FBS level data of this study fall within the framework of continuous longitudinal data and hence can be modeled by use of a parametric linear mixed model. Let *Y*_*ij*_ denote the FBS level of the *i*th patient observed at time *t*_*ij*_, *i*=1,…,*n* and *j*=1,…*m*_*i*_. The parametric linear mixed model can be expressed as

1 $$ Y_{ij} = \sum\limits_{k=0}^{p} \beta_{k}\,t_{ij}^{k} + \sum\limits_{l=1}^{L} \theta_{l}\,x_{ijl} + \sum\limits_{u=0}^{q} b_{u_{i}}\,t_{ij}^{u} + \varepsilon_{ij}.  $$

That is, the population level mean response is modeled as a polynomial function of time, *t*_*ij*_, a linear function of covariates *x*_*ijl*_, *l*=1,…,*p* where some of them may be time-varying covariates or interaction effects each has corresponding regression parameter coefficient *θ*_*l*_, a function of subject-specific random coefficient terms and measurement error *ε*_*ij*_. The coefficients *β*_*k*_, *k*=1,…,*p* and *θ*_*l*_, *l*=1,…,*L* are fixed effect parameters and $b_{u_{i}}$, *u*=0,…,*q* are subject-specific random coefficients. It is assumed that $b_{u_{i}} \sim \mathcal {N}\left (0, \sigma _{b_{u}}^{2}\right)$, $\varepsilon _{ij} \sim \mathcal {N}\left (0, \sigma _{e}^{2}\right)$, $cov\left (b_{u_{i}}, b_{u_{i'}}\right) = \sigma _{b_{u} b_{u}'}$ and $cov\left (b_{u_{i}}, \varepsilon _{ij}\right) = 0$. We have examined models for *p*=2 which represents quadratic polynomial and $b_{u_{i}}$ with *u*=0,1,2 represent a subject-specific random intercept, slope and quadratic coefficients, respectively for selection of a variance-covariance structure (see Table 1). The variance profile plot of FBS level shows (for the sake of brevity this plot is not reported) the variance changes overtime, therefore to allow for more flexibility to estimate between subject variability we have considered the above three variance-covariance structures.

**Table 1:** Linear mixed models for selection of variance-covariance structure for FBS level, JUSH, September 2011 - June 2014

Subject-specific random effect	Linear mixed model
M_1_: Random intercept	$Y_{ij} = \beta _{0} + \beta _{1}\,t_{ij} + \beta _{2}\,t_{ij}^{2} + \sum _{l=1}^{L} \theta _{l}\,x_{ijl} + b_{0_{i}} + \varepsilon _{ij}$
M_2_: Linear random effects	$Y_{ij} = \beta _{0} + \beta _{1}\,t_{ij} + \beta _{2}\,t_{ij}^{2} + \sum _{l=1}^{L} \theta _{l}\,x_{ijl} + b_{0_{i}} + b_{1_{i}}\,t_{ij} + \varepsilon _{ij}$
M_3_: Quadratic random effects	$Y_{ij} \,=\, \beta _{0} \,+\, \beta _{1}\,t_{ij} \,+\, \beta _{2}\,t_{ij}^{2} \,+\, \sum _{l=1}^{L} \theta _{l}\,x_{ijl} \,+\, b_{0_{i}} \,+\, b_{1_{i}}\,t_{ij} \,+\, b_{2_{i}}\,t_{ij}^{2} \,+\,\varepsilon _{ij}$

In Table 1, for instance the subject-specific random intercept $b_{0_{i}}$ in the quadratic random effects model (M_3_) is considered to capture correlation of the FBS level measurements over time within the patient and it is assumed that subject-specific random slopes for linear as well as for quadratic time effects to capture different evolution of FBS level over time. Note that these subject-specific random structures are different for each patient.

#### Tests for zero variance components

Adequate variance-covariance structure is essential to obtain valid model based inferences for the fixed effects or for parameters in the mean structure of the model [18]. Over-parametrization of the variance-covariance structure leads to inefficient estimation and potentially poor assessment of standard errors for the estimation of the mean structure, i.e. fixed effects, whereas a too restrictive specification invalidates inferences about the mean response profile when the assumed structure does not hold.

The likelihood ratio test for testing, for example $H_{0}: \sigma ^{2}_{b_{0}} = 0$ versus $H_{1}: \sigma ^{2}_{b_{0}} > 0$ for model M_1_, has an asymptotic $0.5\,\chi ^{2}_{0} + 0.5\,\chi ^{2}_{1}$ mixture distribution under *H*_0_ [19], if the vector of FBS level can be divided into a large number of independent and identically distributed sub-vectors both under *H*_0_ and *H*_1_. However, this assumption usually does not hold, for example in linear mixed models or for unbalanced data [20–22]. Note that the FBS level data are unbalanced in the sense that all patients do not have equal number of measurements, hence the independent and identically distributed assumption can be violated in the linear mixed models used in this paper. Therefore, we used the exact finite sample null distribution of the restricted likelihood ratio test (RLRT) statistic derived by Crainiceanu and Ruppert [22] to test a zero random effect variance in M_1_. However, since models M_2_ and M_3_ contain more than one random effect, the tests for a zero random effect variance in these models were done using the exact finite sample null distribution of the RLRT statistic derived by Greven et al. [21].

### Semi-parametric mixed effects model

Given the mean profile plots over time in Fig. 1b, imposing parametric functions to describe the mean FBS level evolution may not be easy and also too restrictive [17]. As an alternative, we can model the mean profiles over time with a semi-parametric smooth function, *f*(*t*_*ij*_). Using the *p*th degree truncated power basis, *f*(*t*_*ij*_) can be written as

2 $$ f\left(t_{ij}\right) = \beta_{0} + \beta_{1}\,t_{ij} + \beta_{2}\,t_{ij}^{2} + \ldots + \beta_{p}\,t_{ij}^{p} + \sum\limits_{l=1}^{K} b_{l}\,\left(t_{ij} - \kappa_{l}\right)_{+}^{p},  $$

here *z*_+_=*max*{0,*z*}. The function *f*(*t*_*ij*_) is a combination of fixed effects parameters *β*_0_,*β*_1_,…,*β*_*p*_ and *p*th degree splines evaluated at time *t*_*ij*_ with knots at distinct locations *κ*_1_,*κ*_2_,…,*κ*_*K*_ in the range of *t*_*ij*_ and corresponding coefficients *b*_1_,*b*_2_,…,*b*_*K*_. The function *f*(*t*_*ij*_) can be estimated among others, with penalized splines. The coefficients of spline basis functions *b*_*l*_ are assumed to follow a Gaussian distribution such that $b_{l} \sim \mathcal {N}\left (0, \sigma ^{2}_{b}\right)$, where $\sigma ^{2}_{b}$ is a variance component controlling the smoothness of *f*(*t*_*ij*_). Then, incorporating *f*(*t*_*ij*_) in model (1), the general semi-parametric mixed effects model can be expressed as

3 $$ Y_{ij} = f\left(t_{ij}\right) + \sum\limits_{l=1}^{L} \theta_{l}\,x_{ijl} + \sum\limits_{u=0}^{q} b_{u_{i}}\,t_{ij}^{u} + \varepsilon_{ij}.  $$

### Estimation of parameters

Let $\mathbf {y}_{i} = \left (y_{i1}, y_{i2}, \ldots, y_{{im}_{i}}\right)'$ be the *m*_*i*_×1 vector of responses for the *i*th patient, *i*=1,…,*n*. Under the linear mixed model formulation, model (3) with subject-specific quadratic random effects can be expressed succinctly in matrix form as

4 $$ \mathbf{ y}_{i} = \mathbf{X}_{i}\,\mathbf{\beta} + \mathbf{Z}_{i(f)}\,\mathbf{v} + \mathbf {Z}_{i(u)}\,\mathbf {u}_{i} + \mathbf{e}_{i}  $$

where **β**=(*β*_0_,*β*_1_,…,*β*_*p*_,*θ*_1_,…,*θ*_*L*_)^′^ is a (*p*+*L*+1)×1 vector of fixed effects which is common to the *n* individuals, **X**_*i*_ is an *m*_*i*_×(*p*+*L*+1) design matrix associating **β** to **y**_*i*_, **v**=(*b*_1_,*b*_2_,…,*b*_*K*_) is a *K*-dimensional vector of random coefficients in the summand in Eq. (2), **Z**_*i*(*f*)_ is the *m*_*i*_×*K* matrix for the *p*th-degree spline basis functions, $\textbf {u}_{i} = \left (b_{0_{i}}, b_{1_{i}}, b_{2_{i}}\right)'$ is subject-specific vector of random effects, **Z**_*i*(*u*)_ is an *m*_*i*_×3 design matrix which relates **u**_*i*_ to the response **y**_*i*_ and $\textbf {e}_{i} = \left (e_{1i}, e_{2i}, \ldots, e_{{im}_{i}}\right)'$ is an *m*_*i*_-dimensional vector of within-individual errors. Furthermore, it is assumed that $\textbf {v} \sim \mathcal {N}\left (\textbf {0}, \sigma _{b}^{2}\,\textbf {I}_{K}\right)$, $\textbf {u}_{i} \sim \mathcal {N}(\textbf {0}, \textbf {G})$, $\textbf {e}_{i} \sim \mathcal {N}\left (\textbf {0}, \textbf {R}_{i}\right)$, **v**, **u**_*i*_ and **e**_*i*_ are assumed to be pairwise independent with and between subjects for *i*=1,2,…,*n*. Note that **G** and **R**_*i*_ are 3×3 and *m*_*i*_×*m*_*i*_ variance-covariance matrices, respectively.

The overall model for *n* individuals has the form

$$\mathbf {y} = \mathbf{ X}\,\mathbf{\beta} + \mathbf {Z}\,\mathbf {b} + \mathbf{ e} $$

where

$$\begin{array}{*{20}l} &\textbf{y} = \left (\begin{array}{c} \textbf{y}_{1} \\ \textbf{y}_{2} \\ \vdots \\ \textbf{y}_{n} \end{array} \right) ~~ \textbf{X} = \left (\begin{array}{c} \textbf{X}_{1} \\ \textbf{X}_{2} \\ \vdots \\ \textbf{X}_{n} \end{array} \right),\\ ~~ &\textbf{X}_{i} = \left (\begin{array}{cccccccc} 1 & t_{i1} & t_{i1}^{2} & \ldots & t_{i1}^{p} & x_{i11} & \ldots & x_{i1L} \\ 1 & t_{i2} & t_{i2}^{2} & \ldots & t_{i2}^{p} & x_{i21} & \ldots & x_{i2L} \\ \vdots & \vdots & \vdots & \ddots & \vdots & \vdots & \ddots & \vdots \\ 1 & t_{{im}_{i}} & t_{{im}_{i}}^{2} & \ldots & t_{{im}_{i}}^{p} & x_{{im}_{i}1} & \ldots & x_{{im}_{i}L} \end{array} \right), \end{array} $$

$$\begin{array}{*{20}l} &\textbf{Z} = \left (\begin{array}{ccccc} \textbf{Z}_{1(f)} & \textbf{Z}_{1(u)} & \textbf{0} & \ldots & \textbf{0} \\ \textbf{Z}_{2(f)} & \textbf{0} & \textbf{Z}_{1(u)} & \ldots & \textbf{0} \\ \vdots & \vdots & \vdots & \ddots & \vdots \\ \textbf{Z}_{n(f)} & \textbf{0} & \textbf{0} & \ldots & \textbf{Z}_{n(u)} \end{array} \right),\\ ~~ &\textbf{Z}_{i(u)} = \left (\begin{array}{cccc} 1 & t_{i1} & t_{i1}^{2} \\ 1 & t_{i2} & t_{i2}^{2} \\ \vdots & \vdots & \vdots \\ 1 & t_{{im}_{i}} & t_{{im}_{i}}^{2} \end{array} \right), \end{array} $$

$$\begin{array}{*{20}l} &\textbf{Z}_{i(f)} = \left (\begin{array}{cccc} (t_{i1} - \kappa_{1})_{+}^{p} & (t_{i1} - \kappa_{2})_{+}^{p} & \ldots & (t_{i1} - \kappa_{K})_{+}^{p} \\ (t_{i2} - \kappa_{1})_{+}^{p} & (t_{i2} - \kappa_{2})_{+}^{p} & \ldots & (t_{i2} - \kappa_{K})_{+}^{p} \\ \vdots & \vdots & \ddots & \vdots \\ (t_{{im}_{i}} - \kappa_{1})_{+}^{p} & (t_{{im}_{i}} - \kappa_{2})_{+}^{p} & \ldots & (t_{{im}_{i}} - \kappa_{K})_{+}^{p} \end{array} \right),\\ ~~ &\textbf{e} = \left (\begin{array}{c} \textbf{e}_{1} \\ \textbf{e}_{2} \\ \vdots \\ \textbf{e}_{n} \end{array} \right) \end{array} $$

and $\phantom {\dot {i}\!}\textbf {b} = (b_{1}, b_{2}, \ldots, b_{k}, b_{0_{1}}, b_{1_{1}}, b_{2_{1}}, \ldots, b_{0_{n}}, b_{1_{n}}, b_{2_{n}})'$. Estimation of the coefficients of penalized and unpenalized terms in model (4) was done using a penalized iteratively reweighted least squares (P-IRLS) based on 20 equidistant knots in the range of FBS level and a smoothing parameter selection was done by REML [23].

The correspondence between the penalized spline smoother and the optimal predictor in a mixed model framework enables us to take advantage of the existing methodology for mixed model analysis and the use of mixed model software, such as the function gamm in mgcv R package, for fitting the penalized spline model and the MIXED and GLIMMIX procedures in SAS [24]. This implementation of penalized smoothing in the linear mixed model framework also provides an automated approach to obtain a smoothing parameter and flexibility to extend the models [17].

In this paper, parameters in the fitted models are estimated by restricted maximum likelihood (REML) method because the statistical hypotheses that were considered have the same mean structures between models under the null and alternative hypotheses. Furthermore, maximum likelihood estimators of variance components are biased downward as they do not take into account the degrees of freedom lost in the estimation of fixed effects (e.g. see Ruppert et al. [16]).

### Model selection and inference

The model building process of this work includes selection of suitable variance-covariance structure for random effects, testing whether the inclusion of spline effects in the parametric model improves model fit or not and also selection of covariates. The linear mixed model framework provides a unified approach to do all these [25]. In the parametric cases, the best fitting model can be selected by employing a commonly used selection criteria, Akaike’s Information Criterion (AIC) and Bayesian Information Criterion (BIC) or by a likelihood ratio test. However, since the semi-parametric mixed models that we considered here are differ in both the fixed effects and the nonparametric part, model selection is done via adjusted Akaike’s information criterion, abbreviated AIC _*adj*_, using the effective number of parameters in the model [16, 26]. Let **C**= [**X** **Z**_*f*_] be the design matrix with appropriate fixed effects components and the corresponding smoothing matrix, $\textbf {B} = \left (\begin {array}{cc} \textbf {0} & \textbf {0} \\ \textbf {0} & \textbf {G}^{-1} \end {array} \right)$where **G** is the variance-covariance matrix of random effects used in the model and **R**=*diag*{**R**_1_,**R**_2_,…,**R**_*n*_}, i.e. **R** is the block diagonal variance-covariance matrix of error terms with blocks **R**_*i*_ on the main diagonal and zeros elsewhere. Then the effective number of parameters and AIC _*adj*_ may be computed as

$$E_{p} = trace\left\{\left(\textbf{C}'\,\textbf{R}^{-1}\,\textbf{C}\right)^{-1}\textbf{C}'\,\textbf{R}^{-1}\,\textbf{C} \right\} $$

and AIC _*adj*_=−2 log(*Lik*)+2 *E*_*p*_, respectively. Unlike the marginal AIC which penalizes only for the number of parameters in fixed effects vector and variance components, the penalty of AIC _*adj*_ takes into account the additional parameters introduced into a model via *f*(*t*_*ij*_) or smoothing by including the design matrix **Z**_*f*_ in **C** [17]. Like the marginal AIC, the smaller the AIC _*adj*_ value, the better the model.

Testing whether the inclusion of spline effects in the parametric model improves model fit or not is equivalent to testing $H_{0}: \sigma ^{2}_{b} = 0$ versus $H_{1}: \sigma ^{2}_{b} > 0$. In this paper, due to the second objective of the study, a quadratic penalized spline was added in Eq. (1), therefore neither of the two methods discussed in “Variance-covariance structures and inference” section can be used to test $H_{0}: \sigma ^{2}_{b} = 0$ [27] instead an approximate *F*-test of Hastie and Tibshi [28] was applied. For Hastie and Tibshi approximate *F*-test, residual degrees of freedom for the null and alternative model fits are used in the place of the number of parameters in each model.

### Rate of change over time and simultaneous confidence bands

The change in smoothing function *f*(*t*) overtime, for selected semi-parametric mixed model, can be estimated by taking the derivative of *f*(*t*) with respect to time *t*. For example, let *f*(*t*) be a quadratic penalized spline, that is

$$f(t) = \beta_{0} + \beta_{1}\,t + \beta_{2}\,t^{2} + \sum\limits_{l=1}^{K} b_{l}\,\left(t_{ij} - \kappa_{l}\right)_{+}^{2}. $$

Taking the first derivative with respect to time *t* yields

$$f'(t) = \beta_{1} + 2\,\beta_{2}\,t + 2\,\sum\limits_{l=1}^{K} b_{l}\,\left(t_{ij} - \kappa_{l}\right)_{+}. $$

An estimate of *f*^′^(*t*), denoted $\hat {f}'(t)$, is obtained by substituting the quadratic fit parameter estimates $\hat {\beta }_{1}, \hat {\beta }_{2}$, and $\hat {b}_{1}, \hat {b}_{2}, \ldots, \hat {b}_{K}$. However, the construction of simultaneous confidence bands requires the variance-covariance matrix for the vector of contrasts between the estimated and true parameters for the fixed and random effects. Let **C**= [**X** **Z**_*f*_] be a design matrix containing quadratic time effects and a truncated quadratic basis, **B** is a matrix constructed from variance components corresponding to smoothing, i.e. *Var*(**v**) in model (4). Then, a variance-covariance matrix for the vector of contrasts is given by

$$Var\left (\left[ \begin{array}{c} \hat{\beta} - \beta \\ \hat{\textbf{v}} - \textbf{v} \end{array} \right]\right) \simeq \left(\textbf{C}'\textbf{R}^{-1}\,\textbf{C} + \textbf{B} \right)^{-1} $$

Ruppert et al. [16], where **R** is the block diagonal variance-covariance matrix of error terms defined in “Semi-parametric mixed effects model” section. Let **g**=(*g*_1_,*g*_2_,…,*g*_*T*_) be a grid of equally spaced time points. Define

$$\hat{\textbf{f}}_{g} - \textbf{f}_{g} = \textbf{C}_{g}\,\left(\begin{array}{c} \hat{\beta} - \beta \\ \hat{\textbf{v}} - \textbf{v} \end{array} \right) $$

where **C**_*g*_ is **C** with design matrices **X** and **Z**_*f*_ are evaluated over **g**. Assuming the vector of contrasts have approximately multivariate distribution with mean vector **0** and variance-covariance matrix (**C**^′^**R**^−1^ **C**+**B**)^−1^ [16, 29], i.e.

5 $$ \left(\begin{array}{c} \hat{\beta} - {\beta} \\ \hat{\textbf{v}} - \textbf{v} \end{array} \right) \sim \mathcal{N}\left(\textbf{0}, \left(\textbf{C}'\textbf{R}^{-1}\,\textbf{C} + \textbf{B} \right)^{-1}\right)  $$

a 100 (1−*α*)% simultaneous confidence bands for **f**_*g*_ is given by

6 $$ \hat{\textbf{f}}_{g} \pm h_{(1-\alpha)}\,\textbf{s}_{g}  $$

where $\textbf {s}_{g} \,=\, \left (\widehat {SD}\left (\hat {f}_{g_{1}} \,-\, f_{g_{1}}\right), \widehat {SD}(\hat {f}_{g_{2}} \,-\, f_{g_{2}}), \ldots, \widehat {SD}\left (\hat {f}_{g_{T}} \,-\, f_{g_{T}}\right)\right)'$ with

$${\begin{aligned} \widehat{SD}\left(\hat{f}_{g_{m}} - f_{g_{m}}\right) = \sqrt{\text{the} ~~(m,m)th ~~ \text{diagonal element of}~~ Var\left(\hat{\textbf{f}}_{g} - \textbf{f}_{g}\right)} \end{aligned}} $$

and $Var\left (\hat {\textbf {f}}_{g} - \textbf {f}_{g}\right) = \textbf {C}_{g}\,\left (\textbf {C}'\textbf {R}^{-1}\,\textbf {C} + \textbf {B} \right)^{-1}\,\textbf {C}'_{g}$, and *h*_(1−*α*)_ is the (1−*α*) quantile of

7 $$ \sup\left| \frac{\hat{f}(t) - f(t)}{\widehat{SD}\left\{\hat{f}(t) - f(t)\right\}} \right| \approx \max_{1 \le m\le T} \left| \frac{\left (\textbf{C}_{g}\,\left[ \begin{array}{c} \hat{\beta} - {\beta} \\ \hat{\textbf{v}} - \textbf{v} \end{array} \right]\right)}{\widehat{SD}\left\{\hat{f}(g_{m}) - f(g_{m})\right\} }\right|.  $$

The quantile *h*_(1−*α*)_ can be approximated using simulations. First we simulate from realization of (5) and computation of (7) can be repeated for a large number of times, say *N* times, to obtain $\tilde {h}^{1}_{1-\alpha }, \tilde {h}^{2}_{1-\alpha }, \ldots, \tilde {h}^{N}_{1-\alpha }$. The value with rank *N*×(1−*α*) is used as *h*_1−*α*_.

The proposed semi-parametric mixed models were fitted with the the gamm function available in R package mgcv [29] and the linear mixed models using the lme function available in R package nlme.

## Results

### Patients baseline characteristics

A total of 534 adult diabetic patients were in the study, of which 342 (64.04%) were male, 399 (74.72%) were Type 2 diabetic patients and 417 (78.09%) didn’t have family history of diabetes. The patients mean (SD) age at the first visit (or baseline) was 45.40 (14.62) years and ranges between 18 and 93 years, weight was 62.83 (13.36) kgs and FBS level was 164.72 (86.20) mg/L. There were significant differences of these means between Type 1 and Type 2 diabetic groups (Table 2).The results in Table 2 also show that, at baseline there was a significant association between family history of diabetes and type of diabetes (*p*-value <0.0001). However, the association between patient gender and type of diabetes was nonsignificant (*p*-value = 0.9935). The median (first quartile - third quartile) time between first and last clinic visits of patients was 15.25 (7.25 - 24.75) months and ranged from as few as 0.5 month between visits to as much as 6 months between visits.

**Table 2:** Baseline characteristics of adult diabetic patients in JUSH, September 2011 - June 2014

		Type of diabetes			
Characteristics		Type 1	Type 2	*p*-value	Overall
Gender	Male, N (%)	87 (16.29%)	255 (47.75)	0.9935	342 (64.04%)
	Female, N (%)	48 (8.99%)	144 (26.97%)		192 (35.96%)
Family history	No, N (%)	37 (6.93%)	380 (71.16%)	<0.0001	417 (78.09%)
	Yes, N (%)	98 (18.35%)	19 (3.56%)		117 (21.91%)
Age, mean (SD)		34.55 (11.92)	48.63 (13.78)	<0.0001	45.4 (14.62)
Weight, mean (SD)		58.83 (11.10)	64.02 (13.74)	<0.0001	62.83 (13.36)
FBS, mean (SD)		171.38 (102.39)	162.73 (80.66)	0.0139	164.72 (86.20)

### Parametric mixed models

#### Mean structure

The main interest of this study is to apply semi-parametric mixed models, however for comparison purpose here we start the analysis by fitting parametric mixed models. Scatter plot smoothing was used to examine changes in FBS level over time and also to asses the interactions of each categorical covariate with time [30, 31]. The smoothing plots suggest the changes in FBS can be described by quadratic trend. Furthermore, due to the non-crisscrossing of trends representing Type 1 and Type 2 diabetes groups, and with family history and no family history groups Type × time and Family history × time were not included in the mixed models. However, the trend representing male and female crossing at one time point. Therefore, we start with very general model that includes time (in quadratic form), other fixed effects and the necessary interactions, that is

8 $$ {\begin{aligned} \begin{array}{cc} E(Y_{ij}) & = \beta_{0} + \beta_{1}\,time + \beta_{2}\,time^{2} + \beta_{3}\,Age + \beta_{4}\,Gender + \beta_{5}\,Gender \times time\\ &+\beta_{6}\,Type + \beta_{7}\,F.History + \beta_{8}\,Weight + \beta_{9}\,Weight \times time, \end{array} \end{aligned}}  $$

where Type and F.History represent diabetes type and family history of diabetes, respectively.

### Variance-covariance structure for random effects

The above mean structure fitted with subject-specific random intercepts, linear random time effects and quadratic random time effects. For each of the models, the independent error structure is assumed and the results are given in Table 3.

**Table 3:** Parameter estimates (standard errors, s.e.), *p*-values for associated *t*-tests and model fit criteria, FBS level of diabetes patients in JUSH, September 2011 - June 2014

	Variance-components					
Effects	Random intercept		Linear random effects		Quadratic random effects	
	Estimate (s.e.)	*p*-value	Estimate (s.e.)	*p*-value	Estimate (s.e.)	*p*-value
*Fixed effects*						
Intercept	304.362 (14.616)	<0.0001	306.756 (15.743)	<0.0001	303.139 (15.678)	<0.0001
Age	0.252 (0.183)	0.1693	0.212 (0.179)	0.2362	0.197 (0.179)	0.2699
Gender, Male	-2.605 (5.487)	0.6352	-1.968 (5.983)	0.7424	-2.609 (5.933)	0.6603
Diabetes type, Type 2	-9.758 (8.697)	0.2624	-10.553 (8.814)	0.2317	-10.581 (8.852)	0.2325
Family history, Yes	-12.763 (8.478)	0.1328	-12.335 (8.606)	0.1523	-12.593 (8.643)	0.1457
Time	-4.462 (0.870)	<0.0001	-5.614 (1.071)	<0.0001	-5.549 (1.116)	<0.0001
Time^2^	0.123 (0.018)	<0.0001	0.135 (0.020)	<0.0001	0.153 (0.025)	<0.0001
Weight	-1.981 (0.196)	<0.0001	-1.991 (0.216)	<0.0001	-1.906 (0.215)	<0.0001
Time × Weight	0.016 (0.013)	0.2139	0.032 (0.016)	0.0439	0.025 (0.016)	0.1162
Gender, Male × Time	-0.412 (o.363)	0.2563	-0.482 (0.443)	0.2761	-0.425 (0.444)	0.3390
*Variance components*						
*var*(*b*_0_)	2135.023		2797.766		3352.606	
*var*(*b*_1_)			4.575		40.343	
*var*(*b*_2_)					0.048	
Residual	5023.386		4873.227		4723.609	

The fixed effect estimates were consistent in sign but have slight differences in magnitude across the three different variance-covariance structures. The variables age, gender, diabetes type, family history, and time by weight and gender by time interactions were statistically nonsignificant in all models, except for time by weight interaction where its *p*-value marginally significant for subject-specific random intercept and slope model (i.e. linear random effects model). The covariates that were statistically significant at 5% level, i.e. *Time*, *Time*^2^ and *weight* and the time by weight interaction were retained for the subsequent analysis.

The Crainiceanu and Ruppert [22] RLRT statistic for testing $H_{0}: \sigma ^{2}_{b_{0}}=0$ against $H_{1}: \sigma ^{2}_{b_{0}} > 0$ in model M_1_ takes the value RLRT = 738.24 with *p*-value <0.0001. The large value of the test statistic or a very small *p*-value strongly suggests a rejection of the null hypothesis (i.e. $H_{0}: \sigma ^{2}_{b_{0}}=0$) that no subject-specific random effects should be included in the model. Similar tests were conducted using the exact finite sample null distribution of the RLRT statistic of Greven et al. [21] to test $H_{0}: \sigma ^{2}_{b_{1}}=0$ against $H_{1}: \sigma ^{2}_{b_{1}} > 0$ and $H_{0}: \sigma ^{2}_{b_{2}}=0$ against $H_{1}: \sigma ^{2}_{b_{2}} > 0$ in models M_2_ and M_3_, respectively. The RLRT statistic is 3.944 with *p*-value = 0.0207 for $H_{0}: \sigma ^{2}_{b_{1}}=0$ indicating rejection of the null hypothesis which implies the need for subject-specific random slopes. Whereas the RLRT statistic for $H_{0}: \sigma ^{2}_{b_{2}}=0$ is 0.639 with *p*-value = 0.1859 suggesting a non-rejection of the null hypothesis $H_{0}: \sigma ^{2}_{b_{2}}=0$ which implies no quadratic random effect should be included in the model. Therefore, in the subsequent analysis we use the following parametric linear mixed model, called M_4_:

9 $$ {\begin{aligned} y_{ij} = \beta_{0} + \beta_{1}\,weight + \beta_{2}\,t_{ij} + \beta_{3}\,t_{ij} \times weight + \beta_{4}\,t_{ij}^{2} + b_{0_{i}} + b_{1_{i}}\,t_{ij} + e_{ij}. \end{aligned}}  $$

The analysis results for model M_4_ are presented in Table 4. Except the *time* × *weight* interaction effect, which is marginally non-significant at 5% level, all the fixed effects are highly significant.

**Table 4:** Parameter estimates (standard errors, s.e.) and *p*-values for associated *t*-tests for model M_4_, FBS level of diabetes patients in JUSH, September 2011 - June 2014

Effects	Estimate (s.e.)	*p*-value
*Fixed effects*		
Intercept	302.931 (13.330)	<0.0001
Time	-5.815 (1.061)	<0.0001
Weight	-1.968 (0.212)	<0.0001
Time × Weight	0.031 (0.016)	0.0509
Time^2^	0.134 (0.020)	<0.0001
*Variance components*		
*var*(*b*_0_)	2797.887	
*var*(*b*_1_)	4.601	
Residual	4877.259	

### Semi-parametric mixed model

The observed mean FBS level profile of patients is shown in Fig. 1b. The plot in this figure shows that the linearity assumption is not reasonable. Therefore, the analysis had to account for the longitudinal data structure and the observed nonlinearity of FBS level estimated with smooth effects in the mixed model framework. Given our specific interest in rate of change in FBS level due to clinic interventions, its functional form (over time) can affect the rate of change. Since the rate of change involves taking derivatives of the smooth function *f*(*t*_*ij*_), we choose to use quadratic penalized spline to model the FBS level mean response [31]. Following results from the previous section, we propose the following semi-parametric mixed model with linear random effects structure, called M_5_

10 $$ \begin{aligned} &y_{ij} = \beta_{0} + \beta_{1}\,weight + \beta_{2}\,t_{ij} + \beta_{3}\,t_{ij} \times weight + \beta_{4}\,t_{ij}^{2}\\ &\qquad+ \sum_{l=1}^{K} b_{l}\,(t_{ij} - \kappa_{l})_{+}^{2}+ \sum_{u=0}^{1} b_{u_{i}}\,t_{ij}^{u} + e_{ij}. \end{aligned}  $$

Using appropriately constructed matrices this model can be represented using a matrix notation of “Tests for zero variance components” section. This model is fitted using the random intercept and linear random effects variance structures of the previous section and the results are displayed in Table 5.

**Table 5:** Parameter estimates (standard errors, s.e.), *p*-values for associated *t*-tests and variance components estimates of semi-parametric models under various variance structures, FBS level of diabetes patients in JUSH, September 2011 - June 2014

	Variance structures			
Effects	Random intercept		Linear random effects	
	Estimate (s.e.)	*p*-value	Estimate (s.e.)	*p*-value
*Fixed effects*				
Weight	-1.908 (0.191)	<0.0001	-1.899 (0.212)	<0.0001
Time	28.264 (6.087)	<0.0001	26.742 (6.359)	<0.0001
Time × Weight	0.017 (0.013)	0.1837	0.031 (0.016)	0.0536
Time^2^	0.408 (0.402)	0.3095	0.448 (0.421)	0.2875
s(Time)Fx1	-2971.649 (551.992)	<0.0001	-3014.737 (579.734)	<0.0001
*Variance components*				
Standard deviation				
Intercept	2104.479		2796.166	
Linear			4.814	
Residual	4919.429		4762.647	
s(Time)	13.287	<0.0001	13.939	<0.0001

The results in Table 5 show that the fixed effects estimates were consistent in sign but have slight difference in magnitude in both semi-parametric and parametric mixed models (see Table 4), except for the effect of time where both the sign and magnitude of its coefficient estimates were different in the two models and the effect of "time square" was nonsignificant in the semi-parametric mixed models. Further, the interaction of weight with time was not statistically significant in any of the semi-parametric mixed model. Except for the subject-specific random slope variance component, there is a slight decrease in subject-specific random intercept and residual variance components in the semi-parametric model compared to variance components in the linear mixed model M_4_ (see Table 4).

To compare the two variance structures under the semi-parametric mixed model given in Eq. (10), we computed AIC, BIC and adjusted AIC (see Table 6). Adjusted AIC shows that the semi-parametric mixed model with subject-specific intercepts as well as slopes (or random linear effects) value is smaller than that of the random intercept. Therefore, the semi-parametric model with random linear effects is the preferred model.

**Table 6:** Fit statistics for model M_5_ and M_4_, FBS level of diabetes patients in JUSH, September 2011 - June 2014

Fit statistics					
Variance structure	−2 log(*Lik*)	AIC	BIC	*E*_*p*_	AIC _*adj*_
M_5_					
Random intercept	50538.54	50554.54	50605.63	7.087	50545.627
Random linear	50507.09	50527.09	50590.96	7.260	50514.350
M_4_	50583.51	50601.51	50658.98		

#### Model selection

In this section we are focusing on assessing whether the inclusion of spline effects improves model fit compared to parametric counterpart. This is equivalent to testing $H_{0}: \sigma ^{2}_{b} = 0$ versus $H_{1}: \sigma ^{2}_{b} > 0$ in model M_5_, where $\sigma ^{2}_{b}$ is a variance component controlling the smoothness of

$$f(t_{ij}) = \beta_{0} + \beta_{1}\,t_{ij} + \beta_{2}\,t_{ij}^{2} + \sum_{l=1}^{K} b_{l}\,(t_{ij} - \kappa_{l})_{+}^{2}. $$

The approximate *F*-test statistic for testing the above hypotheses, i.e. quadratic form of *f*(*t*_*ij*_) against a quadratic penalized splines, is 83.63 with *p*-value <0.0001. This strongly suggests a rejection of the null hypothesis $H_{0}: \sigma ^{2}_{b} = 0$. Thus, the shape of the function *f*(*t*_*ij*_) is statistically different from a quadratic trend.

Furthermore, consider the semi-parametric mixed model M_5_ in Eq. (10) with random linear effects variance-covariance structure and the linear mixed model M_4_ in Eq. 9. The fit statistics from fitting these two models are displayed in Table 6. The −2 log(*Lik*), AIC and BIC values indicate a substantial improvement in the fit of M_5_ compared to M_4_, implying model with penalized spline representation of FBS level was preferred over its parametric counterpart.

The overall results show that, out of the models evaluated, FBS level of diabetes patients at the JUSH diabetic clinic during the study period best characterized by a penalized spline model with truncated quadratic basis, with subject-specific random intercept and slope effects and with linear function of weight and time, called the final model, M_6_.

#### Simultaneous confidence band

**Fig. 2:**
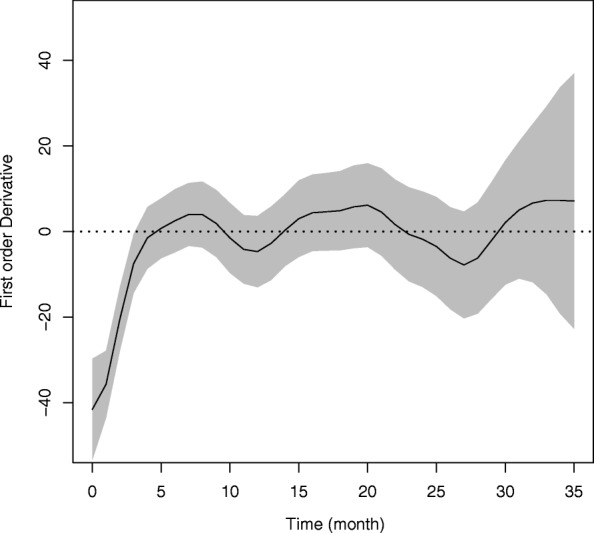
95% simultaneous confidence bands for FBS level of diabetes patients in JUSH, September 2011 - June 2014

The first derivative of mean response function, i.e. $\hat {f}'(.)$, with respect to time was estimated for the final model, M_6_ holding weight constant. The rate of change in mean response of FBS level then investigated using the 95% simultaneous confidence bands for the model. The confidence bands were constructed following the discussion in “Estimation of parameters” section. A gride **g** of time points (0, 35) were defined by increments of one month such that there are *T*=36 equally spaced time points. The resulting simultaneous confidence bands displayed in Fig. 2 where the solid line and shaded region represent, respectively, the mean predicted FBS level and the confidence bands. Visual inspection indicates that on average diabetes patients were able to decrease or control their FBS level, due to JUSH clinic interventions, in the first five months period after their initial visit. However, after month 5, the slope of the curve starts changing it signs, this might imply that patients do actually not follow-up the intervention packages properly or not come to the clinic for treatment due to some unknown reason.

The confidence bands become noticeably wider after 27 months of follow-up period, demonstrating the increased variability. This increase may be due to a smaller number of FBS level recordings being observed at the later period of the study or a potential artifact induced by the spline smoothing [32]. In practice spline smoothing creates a challenge in semi-parametric regression settings through the inherent bias from using truncated basis functions. The confidence bands obtained for FBS level does not account for this function bias. However, this bias could be corrected, e.g. using bootstrapping methods [33].

## Discussion

This study focused on longitudinal data analysis of fasting sugar level of adult diabetic patients at Jimma University Specialized Hospital diabetic clinic using an application of semi-parametric mixed model. The study revealed that the rate of change in FBS level in diabetic patients, due to the clinic interventions, does not continue as a steady pace but changes with time and weight of patients. Furthermore, it clarified the associations between FBS level and some characteristics of adult diabetic patients that weight of a diabetes patient has a significant negative effect whereas patient gender, age, type of diabetes and family history of diabetes did not have a significant effect on the change of FBS level. The result on gender agrees with the findings of [34] where the gender effect on fasting blood glucose level of type 2 diabetes was statistically nonsignificant.

Under the two variance-covariance structures of subject-specific random effects, the semi-parametric mixed models had better fit than their parametric counterparts. This was likely due to the localized splines which captured more variability in FBS level than the linear mixed models. The methodology used in the analysis has implications for clinical monitoring in regular followup of diabetic patients and to assess the effect of intervention packages, such as healthy diet, physical activity and prescribed medicines, because individualized curve may be obtained to follow patient-specific FBS level trends [31].

The main limitation of the study is the limited information on important predictors such as type of interventions including treatment types and nutritional status of a patient that may have influenced the rate of change in FBS level. Due to lack of data on these potential predictors for most of the patients involved in the study, we were unable to include them in the analyses. Therefore, more public health and epidemiology researches are needed to examine the impact of treatments and interventions on population health in general and in particular, people living with diabetes to avoid its complications over time and to identify new risk factors for diabetes.

## Conclusion

In this paper, we demonstrate the use of semiparametric mixed effect model for estimation of the rate of change of fasting blood sugar (FBS) level experienced by patients over time. The proposed method can help a physician in clinical monitoring of diabetic patients and to assess the effect of intervention packages such as healthy diet, physical activity.

## Abbreviations

AIC: Akaike’s information criteria
BIC: Bayesian information criteria
FBS: Fasting blood sugar level
IDF: International diabetes federation
JUSH: Jimma University specialized hospital
P-IRLS: Penalized iteratively reweighted least squares
REML: Restricted maximum likelihood
RLRT: Restricted likelihood ratio tests
SD: Standard deviation
